# Experience and practices of doctors towards pharmacist medication reconciliation service in medical wards: a multicentre study in Perak state Malaysia

**DOI:** 10.1080/20523211.2025.2604308

**Published:** 2025-12-23

**Authors:** Ros Sakinah Kamaludin, Chang Chee Tao, Oh Hoey Lin, Chan Pooi Yee, Teh Wen Yan, Chew Wei Yee, Ng Ling Wei, Teoh Lee Rhui

**Affiliations:** a Pharmacy Department, Hospital Raja Permaisuri Bainun, Ministry of Health Malaysia, Perak, Malaysia; b Clinical Research Centre, Hospital Raja Permaisuri Bainun, Institute for Clinical Research, National Institute of Health, Ministry of Health, Ipoh, Perak, Malaysia; c Pharmacy Department, Hospital Slim River, Ministry of Health Malaysia, Perak, Malaysia; d Pharmacy Department, Hospital Tapah, Ministry of Health Malaysia, Perak, Malaysia; e Pharmacy Department, Hospital Selama, Ministry of Health Malaysia, Perak, Malaysia; f Pharmacy Department, Hospital Parit Buntar, Ministry of Health Malaysia, Perak, Malaysia; g Pharmacy Department, Hospital Sungai Siput, Ministry of Health Malaysia, Perak, Malaysia

**Keywords:** Medication reconciliation, doctors, ward pharmacist, pharmacy service, experience, practice

## Abstract

**Background:**

Medication reconciliation is a key responsibility of ward pharmacists, who work closely with doctors. However, little is known about doctors’ practices in the administration of this service.

**Methods:**

A cross-sectional survey was conducted with doctors from the medical wards of 14 Perak government hospitals. A validated self-administered questionnaire was used to assess doctors’ demographics, experiences with pharmacist-led medication reconciliation services (13 items) and practices in administering medication reconciliation-related information (8 items). Responses were measured on a 5-point Likert scale.

This study explored doctors’ experiences with and utilisation of medication reconciliation services provided by pharmacists in medical wards. The independent *t*-test and Kruskal–Wallis test were performed to evaluate the associations between the doctor's sociodemographic backgrounds with their experiences and practices towards pharmacist-led medication reconciliation services.

**Results:**

A total of 209 doctors responded, with more than half of them being female (60.8%) and medical officers (76.6%). Most respondents agreed that ward pharmacists play an integral role (97.6%), perform quality reconciliation (97.2%), and reduce medication errors (97.1%) during medication reconciliation. The majority frequently expected the pharmacists to provide medication history assessments (90.0%), relied on their medication history assessments during prescribing at admission (88.6%) and discharge (89.0%), and collaborated on reconciliation issues (91.8%). Higher practice scores were observed among medical officers (3.89 ± 0.63, *p* = 0.01) and those aged >30 years (3.9 ± 0.46, *p* = 0.04), and those working in wards with ≥30 beds (3.94 ± 0.48, *p* = 0.02).

**Conclusion:**

Doctors demonstrated positive experiences and practices regarding pharmacist-led medication reconciliation services provided by ward pharmacists. These findings suggest that doctors acknowledge the role of pharmacists in medication reconciliation services.

## Background

The World Health Organization (WHO) defines medication reconciliation as ‘the formal process in which healthcare professionals partner with patients to ensure accurate and complete medication information transfer at interfaces of care’. The steps of medication reconciliation consist of identifying and resolving any pharmacotherapy discrepancies, which include the medication dose, frequency, route, omission and commission for therapeutic purposes (Hung et al., 2021).

Pharmacist-led medication reconciliation has been implemented in healthcare settings in many countries over the years, where studies have shown that it improved patients’ care as well as clinical and economic outcomes (Farajallah et al., 2024; Hung et al., 2021; Patel et al., 2019). Pippins et al. (2005) reported that the unintentional discrepancies detected by pharmacists in medical wards accounted for 12% of the total discrepancies detected during medication reconciliation, the majority of which occurred during admission medication history assessment and the rest during patients’ discharge. In addition, medication reconciliation performed by a pharmacist has been shown to reduce patients’ readmission rates and reduce emergency department visits (Patel et al., 2019).

Despite the benefits of pharmacist-led medication reconciliation, it is not widely employed in health institutions for many reasons, including resource limitations (Lesselroth et al., 2022). A survey in the United States Veteran Affairs Department revealed that the majority of healthcare staff disclosed their inability to implement medication reconciliation services, whereas among the barriers reported were the inability to include the new service in their current workflow (Lesselroth et al., 2022). A survey in Kuwait government hospitals revealed low awareness among doctors and pharmacists of their institution's medication reconciliation policy (Lemay et al., 2019). Aires-Moreno et al. (2020) analysed the perceptions of nurses, pharmacists and prescribers about medication reconciliation in Brazil teaching hospitals where both pharmacists and prescribers cited themselves as professionals who were the most responsible in performing medication reconciliation.

Ward pharmacy service was initiated in Malaysian government hospitals in 2004, where medication reconciliation was part of the daily workflow (Ng et al., 2021; Pharmaceutical Services Division, State Health Department Perak, 2022). The medication reconciliation process starts with obtaining the patient's pre-admission medication history, in which the pharmacists produce the best-possible medication history list by obtaining information from more than one source, including the patient's or caretaker's interview, the patient's prescription, the patient's medication physical stock and healthcare electronic medical and medication records (Pharmaceutical Services Division, State Health Department Perak, 2023). The number of medication history assessment forms (CP1) documented during patients’ admission to Perak state government hospitals was 82230 in 2022 (Pharmaceutical Services Division, State Health Department Perak, 2022). Throughout hospitalisation, patients’ pharmacotherapy is monitored and documented using the ‘pharmacotherapy review’ (CP2) form, where the medication list is actively monitored (Pharmaceutical Services Division, State Health Department Perak, 2023). Finally, the ward pharmacists screen the patients’ discharge prescriptions and communicate discrepancies with the doctors to ensure that the information is accurate and complete based on the patients’ current clinical condition.

Although medication reconciliation services have been a fundamental duty of ward pharmacists in health care settings in the Malaysian government over the last decade, where they have worked closely with doctors in communicating patients’ most up-to-date medication lists, little is known about doctors’ familiarity with this service. Therefore, this study aimed to explore the experience of doctors with pharmacists’ medication reconciliation services and the practices of doctors in administering medication reconciliation-related information and interventions performed by pharmacists in medical wards, and to determine the associations of these variables with doctors’ sociodemographic background.

## Methods

### Study design and setting

This study was a questionnaire-based, cross-sectional survey conducted from 20th September to 22nd November 2023 in 14 government hospitals in Perak state, Malaysia. All contract or permanently employed doctors of the Ministry of Health, Malaysia, who had experience working with ward pharmacists in the medical wards of Perak government hospitals were included. Doctors who worked in a mental institution were excluded to avoid response bias.

### Sample size

By using a sample size formula for a prevalence study with an estimation of a potential population of 360 doctors consisting of physicians, medical officers and house officers who work in medical wards, a confidence interval of 95% and margin of error of 5%, with a proportion of 50% of doctors with positive experience and practice towards pharmacist medication reconciliation services, using the Raosoft sample size calculator, 187 participants were needed. The projected sample size of the respondents was 209 participants after considering an estimation of a 90% completion rate.

### Data collection and instrumentation

The self-administered questionnaire was developed on the basis of a literature review on the context and impact of pharmacist-led medication reconciliation (Agency for Healthcare Research and Quality, USA, WHO, and the Commonwealth Fund, 2016; American Society of Health-System Pharmacists, 2013; Farajallah et al., 2024; Hung et al., 2021; Patel et al., 2019). The questionnaire comprises three domains: (1) demographic profile; (2) experience of doctors in medication reconciliation services by ward pharmacists (13 questions); and (3) practices of doctors in administering medication reconciliation-related information and interventions performed by pharmacists (8 questions). The responses were measured using 5-point Likert scale.

The questionnaire underwent face validation and content validation by two senior clinical pharmacists in ward pharmacy services. The questionnaire was then pre-tested on 10 respondents to ascertain feasibility. The reliability and validity of the questionnaire were subsequently confirmed through a pilot study conducted with 32 respondents. The Cronbach's alpha value (*α*) was 0.985 and 0.724 for experience domain and practice domain respectively.

Data collection was conducted with support from the Perak Pharmaceutical Services Division (BPF) and appointed ward pharmacist representatives across the involved hospitals. An online training session was conducted to brief all the data collectors on their role and responsibilities in this study and to clarify any issue pertaining to the data collection form. At least one representative from each involved hospital has participated. If the data collectors encounter any missing data during data collection, they can discuss directly with the primary or site investigators.

After permission was obtained from all the study sites, a Google form link to the questionnaire was distributed to the target respondents via email and WhatsApp Messenger. The link first directed participants to an informed consent page, where they could indicate their consent to participate. Those who agreed proceeded to the questionnaire, whereas those who declined exited the session. During the data collection period, two gentle reminder communications were sent by data collector, either via verbal or text to the participants to encourage their participations.

All the responses were securely stored in an email inbox accessible only to the study investigators.

### Data analysis

Data analysis was performed using SPSS version 21. The demographic data of the respondents were analysed descriptively, and all of the variables were categorised and represented as frequencies and percentages. Data normality was tested using histogram before inferential analysis was performed in both the experience and the practice domain. Independent *t*-test was used for two categorical variables and Kruskal–Wallis test was used for three categorical variables. A *p*-value of less than 0.05 was considered statistically significant. For the experience domain, item number 2 was negatively scored, where a score of 1 was replaced with 5 and vice versa.

## Results

A total of 209 doctors completed the questionnaire, of whom the majority (*n* = 127, 60.8%) were female and medical officers (*n* = 160, 76.6%) (Table 1). The respondent age groups were 54.1% and 45.9% for 21–30 and 31–50 years old respectively. Around 76.6% of the respondents (i = 160) are working as medical officer, followed by specialist (*n* = 28, 13.4%) and house officer (*n* = 21, 10%). Meanwhile, more than half of them (*n* = 124, 59.3%) had worked in the medical ward for more than 1 year, and three out of five (*n* = 124, 60.5%) had worked in ward with 30 or more beds.

**Table 1.** Demographic characteristics of respondents (*n* = 209).

Demographic	*n* (%)
Gender	
Male	82 (39.2)
Female	127 (60.8)
Age group (years)	
Median (year)	30
21- 30	113 (54.1)
31- 50	96 (45.9)
Designation of job	
House officer	21 (10.0)
Medical officer	160 (76.6)
Specialist	28 (13.4)
Working experience	
<1 month	15 (7.2)
1 month to 1 year	70 (33.5)
>1 year	124 (59.3)
Working ward bed number*	
<30	81 (39.5)
≥30 beds	124 (60.5)

*n = 205 due to incomplete data.

The majority of the respondents agreed and strongly agreed with all of the questions in the experience domain (Table 2). All of the items in this domain recorded the answers of ‘agree’ and ‘strongly agree’ in 90% of the respondents, except for item number 9: ‘Ward pharmacist-led medication reconciliation aids in making appropriate diagnosis and treatment planning’ (89%). More than half of the respondents strongly agreed that ward pharmacists identify and reduce medication errors during medication reconciliation (69.8%), play an integral role in medication reconciliation (66.5%), perform good quality medication reconciliation (60.8%), and show credibility in medication reconciliation (58.4%).

**Table 2.** Experience of doctors towards pharmacist medication reconciliation service in medical ward (*n* = 209).

No	Experiences	Response *n* (%)				
		Strongly disagree	Disagree	Neutral	Agree	Strongly agree
1	Ward pharmacists show credibility in medication reconciliation	4 (1.9)	0 (0)	5 (2.4)	78 (37.3)	122 (58.4)
2	Ward pharmacists have an integral role in medication reconciliation	3 (1.4)	0 (0)	2 (1.0)	65 (31.1)	139 (66.5)
3	Ward pharmacists perform good quality medication reconciliation	3 (1.4)	0 (0)	3 (1.4)	76 (36.4)	127 (60.8)
4	Ward pharmacists identify clinically important unintentional medication discrepancies during medication reconciliation	4 (1.9)	0 (0)	10 (4.8)	77 (36.8)	118 (56.5)
5	Ward pharmacists identify and reduce medication error during medication reconciliation	4 (1.9)	0 (0)	2 (1.0)	57 (27.3)	146 (69.8)
6	Ward pharmacists identify and reduce drug–drug interaction during medication reconciliation	4 (1.9)	1 (0.5)	10 (4.8)	81 (38.7)	113 (54.1)
7	Ward pharmacists identify drug allergy and prevent potential patient's harm during medication reconciliation	3 (1.4)	0 (0)	9 (4.3)	78 (37.3)	119 (57.0)
8	Ward pharmacists identify polypharmacy and prevent adverse drug reaction during medication reconciliation	3 (1.4)	0 (0)	16 (7.7)	92 (44.0)	98 (46.9)
9	Ward pharmacist-led medication reconciliation aids in making appropriate diagnosis and treatment planning	3 (1.4)	0 (0)	20 (9.6)	87 (41.6)	99 (47.4)
10	Ward pharmacist-led medication reconciliation promotes appropriate prescribing	3 (1.4)	0 (0)	8 (3.8)	88 (42.1)	110 (52.6)
11	Ward pharmacists encourage patient to bring patients’ medication to ward during medication reconciliation, resulted in minimised medication cost	3 (1.4)	3 (1.4)	15 (7.2)	90 (43.1)	98 (46.9)
12	Ward pharmacists educate patients and their caregivers during medication reconciliation, therefore increase patients’ understanding and compliance on their medication	4 (1.9)	1 (0.5)	7 (3.3)	75 (35.9)	122 (58.4)
13	Ward pharmacist-led medication reconciliation enables the sharing of information obtained from patients, avoiding unnecessary overload or duplication of tasks	4 (1.9)	1 (0.5)	5 (2.4)	94 (45.0)	105 (50.2)

Table 3 shows that the majority of the respondents (90.0%) frequently expected ward pharmacists to provide a medication history assessment form (CP1) when they reviewed newly admitted patients in the ward. However, most of them (88.5%) would still frequently ask for patients’ medication history upon patients’ admission, independently of ward pharmacists’ medication history assessment, and only 39.2% of them would skip medication history assessment when it was performed by a pharmacist. The majority of the respondents frequently relied on the pharmacist medication history assessment form during patients’ medication prescribing upon admission (88.6%) and upon discharge (89.0%). Most of the respondents (91.8%) frequently worked together with ward pharmacists on resolving issues pertaining to medication reconciliation in a systematic workflow, and 93.3% frequently accepted the intervention provided by ward pharmacists. More than half of the respondents (71.8%) frequently documented medication reconciliation-related information or interventions provided by ward pharmacists.

**Table 3.** Practices of doctors in administering the medication reconciliation-related information and intervention done by pharmacist (*n* = 209).

No	Practices	Response *n* (%)				
		Never *n* (%)	Rarely *n* (%)	Occasionally *n* (%)	Frequently *n* (%)	Very frequently *n* (%)
1	How often do you expect the ward pharmacists to provide medication history assessment form (CP1) when you review newly admitted patients in the ward?	0 (0)	2 (1.0)	19 (9.1)	99 (47.4)	89 (42.6)
2	How often do you ask for patients’ medication history upon patients’ admission, independently of ward pharmacists medication history assessment?	0 (0)	2 (1.0)	22 (10.5)	91 (43.5)	94 (45.0)
3	How often do you skip assessing patients’ medication history, when the ward pharmacists have performed the medication reconciliation?	20 (9.6)	38 (18.2)	69 (33.0)	54 (25.8)	28 (13.4)
4	How often do you refer to the CP1 (medication history assessment form) when you prescribe the patients’ medication on admission?	2 (1.0)	7 (3.3)	15 (7.2)	58 (27.8)	127 (60.8)
5	How often do you refer to the CP1 (medication history assessment form) when you prescribe the patients’ medication on discharge?	1 (0.5)	7 (3.3)	15 (7.2)	65 (31.1)	121 (57.9)
6	How often do you work together with the ward pharmacists on resolving issues pertaining to medication reconciliation in a systematic workflow in the ward?	1 (0.5)	2 (1.0)	14 (6.7)	82 (39.2)	110 (52.6)
7	How often do you accept the intervention related to medication reconciliation provided by ward pharmacists?	0 (0)	1 (0.5)	13 (6.2)	99 (47.4)	96 (45.9)
8	How often do you document the medication reconciliation-related information or intervention provided by ward pharmacists?	6 (2.9)	17 (8.1)	36 (17.2)	88 (42.1)	62 (29.7)

The practice of doctors in administering medication reconciliation-related information and interventions performed by pharmacists was significantly associated with their job designation, age group and number of beds in the medical ward in which they were working (Table 4). The practice mean score was significantly higher for medical officers (mean (SD) = 3.89 (0.63), *p* = 0.01), those aged more than 30 years old (mean (SD) = 3.9, (0.46), *p* = 0.04), and those who worked in medical wards with 30 or more beds (mean (SD) = 3.94 (0.48), *p* = 0.02). For the association between the doctors’ practice and the number of beds in the ward they work in, only 205 responses are included for analysis due to four incomplete data entries.

**Table 4.** Association between experience and practice with demographic characteristics.

Demographic (*n* = 209)		*n* (%)	Experience		Practice	
			Mean (SD)	*p-*value	Mean (SD)	*p-*value
Gender	Male	82 (39.2)	4.47 (0.50)	0.91	3.76 (0.46)	0.69
	Female	127 (60.8)	4.46 (0.70)		3.89 (0.46)	
Age group	21 - 30 years old	113 (54.1)	4.48 (0.56)	0.64	3.78 (0.46)	0.04
	31 - 50 years old	96 (45.9)	4.44 (0.70)		3.9 (0.46)	
Designation of job	House officer	21 (10.0)	4.23 (0.50)*	0.06**	3.5 (0.63)*	0.01**
	Medical officer	160 (76.6)	4.63 (0.90)*		3.89 (0.63)*	
	Specialist	28 (13.4)	4.42 (0.90)*		3.88 (0.50)*	
Working experience	Less than 1 month	15 (7.2)	4.27 (1.00)*	0.15**	3.75 (0.56)*	0.05**
	1 month to 1 year	70 (33.5)	4.38 (0.85)*		3.75 (0.63)*	
	More than 1 year	124 (59.3)	4.69 (0.90)*		3.89 (0.63)*	
Working wad bed number***	< 30 beds	81 (39.5)	4.45 (0.48)	0.72	3.78 (0.45)	0.02
	≥ 30 beds	124 (60.5)	4.49 (0.84)		3.94 (0.48)	

All statistical analyses were performed using independent *t*-test unless otherwise stated.

*median (interquartile range).

**Kruskal–Wallis test.

****n = 205 due to incomplete data.

## Discussion

This study revealed that the majority of the doctors had positive experiences pertaining to medication reconciliation services carried out by ward pharmacists in medical wards. They acknowledged that ward pharmacists have a fundamental role and credibility in medication reconciliation, and perform good quality medication reconciliation. Abudleek et al. (2022) reported that 94.6% of doctors agreed that clinical pharmacists play an integral role in the medical team. Pharmacists have distinct knowledge, expertise, and abilities to establish and preserve the medication reconciliation process in hospitals (American Society of Health-System Pharmacists, 2013). Pharmacists should be the manager of the process and serve as patient advocates throughout the transition of care (Splawski & Minger, 2016).

This study revealed that the majority of the doctors recognised that medication reconciliation carried out by a pharmacist identified and reduced medication errors and medication discrepancies. This shows that the pharmacists in medical wards succeeded in achieving the prominent purpose of medication reconciliation (American Society of Health-System Pharmacists, 2013; Mekonnen et al., 2016). This confirmed the findings of previous studies, which concluded that medication reconciliation by pharmacists has a positive impact on patient care by preventing potential harm due to unintentional medication discrepancies (Splawski & Minger, 2016; TS Chen, 2021; Dong et al., 2022). Minimising medication errors provides safe medication use and improves patients’ therapeutic outcomes (Abudleek et al., 2022; Dong et al., 2022; Splawski & Minger, 2016). This is one of the driving factors of the demand for clinical pharmacist services to be implemented in the inpatient setting (Dong et al., 2022).

Our findings indicated that the majority of the doctors in this study also recognised that pharmacist-led medication reconciliation approached drug-related issues through the identification of patients’ drug allergies, drug–drug interactions and polypharmacy, prevented adverse events and potential patient harm. These findings are aligned with other studies which also reported further reduction of adverse drug events postdischarge and re-hospitalisation rates with pharmacist-led medication reconciliation (Abudleek et al., 2022; Karapinar-Çarkit et al., 2012). Meantime, pharmacist-led medication reconciliation also brings additional cost savings, up to 55 euros per patient at 6 months’ post discharge, as shown by a study in a pulmonology department by Karapinar-Çarkit et al. (2012).

The majority of the doctors in the study admitted that pharmacist-led medication reconciliation empowered information sharing, aiding them in appropriate diagnosis and treatment planning, and subsequently avoiding unnecessary duplication of tasks. This finding surpassed that of Hamadi et al. (2015) who reported that only approximately half of the doctors agreed for pharmacists to assist them in planning drug therapy treatment for patients. The discrepancies could be explained by the frequency of interactions between the doctors and the pharmacists in the study by Hamadi et al. (2015) where they reported that the doctors rarely interact with the pharmacists, and the main purpose of the interaction was to inquire about medication availability. Moreover, in our study the medical ward pharmacists communicated with the doctors on a daily basis and are very involved in patient care.

The doctors also agreed that the ward pharmacist educated patients in the ward during the medication reconciliation process. This finding concurred with the highlighted role of pharmacists in patients’ education (Alipour et al., 2018; Hamadi et al., 2015). Pharmacists are highly suited to educate patients on the appropriate and safe use of medications, including their indications, dosages, administration methods, potential adverse effects, and drug interactions. Pharmacist-led medication counselling has been shown to enhance patient adherence to prescribed treatments (Rangkuti et al., 2018; Sayantan et al., 2020). A study conducted in an Indonesian hospital demonstrated that pharmacist-provided counselling significantly improved medication adherence in dyslipidaemia patients, as measured by objective tools (Rangkuti et al., 2018).

In addition, patients’ education carried out by ward pharmacists can establish strong professional relationship between patients and pharmacists (Sayantan et al., 2020). Approximately 90% of the participants agreed that by encouraging patients to bring their medications to the ward, ward pharmacists-led medication reconciliation helped in minimise medication costs. This ensures that patients’ medications are readily available when there is continuation of regular prescription during admission and that there is a reduced number of missing doses. The benefits of patients bringing their medication include significant cost savings, assisting adherence assessment by performing pill counts, and ensuring proper medication disposal when any of them are de-prescribed upon discharge (Nasir et al., 2022).

This study revealed that the majority of the doctors relied on ward pharmacists to perform medication reconciliation for newly admitted patients in medical wards. The Malaysia Ministry of Health government health facility's pharmacies, including those in Perak state, utilised the Pharmacy Information System (PhIS), which is a computerised system in handling the patients’ medication records. PhIS system accessibility enables pharmacists to trace patients’ medication records and document organised medication history assessments. However, a significant proportion of doctors in medical wards continue to independently obtain patients’ medication histories, even when this information is readily available. This may be attributed to the fact that a thorough medical assessment requires a complete medication history, and many doctors prefer to gather this information directly from patients, despite the medication reconciliation provided by pharmacists (Jonathan et al., 2024).

Doctors view themselves as the main healthcare provider throughout inpatient care steps, which the medication history assessment was traditionally performed prior to the introduction of clinical pharmacy services (Zaal et al., 2020). Regardless, encouragingly, the majority of the doctors in this study referred to the medication reconciliation information when prescribing and accepting interventions provided by pharmacists while working together in the ward. This showed their trust in the service and manifested good collaborative relationships between these two professions (Abudleek et al., 2022). Pharmacists who carry out medication reconciliation during patients’ admission and discharge target the most vulnerable points, as these are the points where unintentional medication discrepancies and information miscommunication could occur (American Society of Health-System Pharmacists, 2013; Mekonnen et al., 2016). As a result, assisted healthcare teams can ensure the safe and smooth transition-of-care (American Society of Health-System Pharmacists, 2013; Mekonnen et al., 2016).

Doctors who were more than 30 years old with the majority of medical officers had more favourable practices toward pharmacist medication reconciliation services. We believe that these groups of doctors are the ones most commonly encountered by ward pharmacists in medical wards. Work experience enhances interprofessional communication and collaborative practice (Mekonnen et al., 2016). Naturally, the more interaction there are, the greater the potential for doctors to recognise the role of pharmacists (Mekonnen et al., 2016). The practice of doctors administering medication reconciliation and interventions was significantly associated with the number of beds in their ward. High occupancy wards were associated with greater number of patients and greater workloads. Medication reconciliation by pharmacists helps doctors spend less time on patients’ medication history taking and hence reduces doctors’ workload.

The study has strengths and limitations. To our knowledge, this is the first study in a Malaysian government hospital setting assessing doctors’ experience with and practices related to pharmacist medication reconciliation services. However, this study was limited to a single state in Malaysia, which may affect the generalisability of the findings to other regions and patient populations. Additionally, the reliance on self-reporting introduces the potential for recall bias.

## Conclusion

Doctors in Perak government hospitals have favourable experiences and practices related to the medication reconciliation services provided by pharmacists in medical wards. The findings suggest that doctors acknowledge the potential benefits of pharmacist-led medication reconciliation services, which may contribute to improved patient outcomes, indicating that the pharmacist's role is recognised as an important part of the healthcare team.
